# Accurate and reproducible invasive breast cancer detection in whole-slide images: A Deep Learning approach for quantifying tumor extent

**DOI:** 10.1038/srep46450

**Published:** 2017-04-18

**Authors:** Angel Cruz-Roa, Hannah Gilmore, Ajay Basavanhally, Michael Feldman, Shridar Ganesan, Natalie N.C. Shih, John Tomaszewski, Fabio A. González, Anant Madabhushi

**Affiliations:** 1 Universidad Nacional de Colombia, Bogota, Colombia; 2 Universidad de los Llanos, Villavicencio, Colombia; 3 University Hospitals Case Medical Center, Cleveland, OH, USA; 4 Inspirata Inc., Tampa, FL, USA; 5 Hospital of the University of Pennsylvania, Philadelphia, PA, USA; 6 Cancer Institute of New Jersey, New Brunswick, NJ, USA; 7 University at Buffalo, The State University of New York, Buffalo, NY USA; 8 Case Western Reserve University, Cleveland, OH, USA

## Abstract

With the increasing ability to routinely and rapidly digitize whole slide images with slide scanners, there has been interest in developing computerized image analysis algorithms for automated detection of disease extent from digital pathology images. The manual identification of presence and extent of breast cancer by a pathologist is critical for patient management for tumor staging and assessing treatment response. However, this process is tedious and subject to inter- and intra-reader variability. For computerized methods to be useful as decision support tools, they need to be resilient to data acquired from different sources, different staining and cutting protocols and different scanners. The objective of this study was to evaluate the accuracy and robustness of a deep learning-based method to automatically identify the extent of invasive tumor on digitized images. Here, we present a new method that employs a convolutional neural network for detecting presence of invasive tumor on whole slide images. Our approach involves training the classifier on nearly 400 exemplars from multiple different sites, and scanners, and then independently validating on almost 200 cases from The Cancer Genome Atlas. Our approach yielded a Dice coefficient of 75.86%, a positive predictive value of 71.62% and a negative predictive value of 96.77% in terms of pixel-by-pixel evaluation compared to manually annotated regions of invasive ductal carcinoma.

Detection of tumor cells in a histologic section is the first step for the pathologist when diagnosing breast cancer (BCa). In particular, tumor delineation from background uninvolved tissue is a necessary prerequisite for subsequent tumor staging, grading and margin assessment by the pathologist^1^. However, precise tumor detection and delineation by experts is a tedious and time-consuming process, one associated with significant inter- and intra-pathologist variability in diagnosis and interpretation of breast specimens^2^,^3^,^4^,^5^,^6^.

Invasive breast cancers are those that spread from the original site (either the milk ducts or the lobules) into the surrounding breast tissue. These comprise roughly 70% of all breast cancer cases^7^,^8^, and they have poorer prognosis compared to the *in-situ* sub-types^7^. Isolation of invasive breast cancer allows for further analysis of tumor differentiation via the Bloom-Richardson and Nottingham grading schemes, which estimate cancer aggressiveness by evaluating histologic characteristics including: tubule formation, nuclear pleomorphism and mitotic count^1^. Therefore, an automated and reproducible methodology for detection of invasive breast cancer on tissue slides could potentially reduce the total amount of time required to diagnose a breast case and reduce some of this inter- and intra-observer variability^9^,^10^.

Digital pathology refers to the process of digitization of tissue slides. The process of slide digitization could enable more efficient storage, visualization, and pathologic analysis of tissue slides and could potentially improve overall efficiency of routine diagnostic pathology workflow^11^.

Quantitative histomorphometry refers to the application of computational image analysis and machine learning algorithms to identify and characterize disease patterns on digitized tissue slides^12^. In the context of breast cancer pathology, a number of computational imaging approaches have been recently applied for problems such as (i) detection of mitoses^13^,^14^,^15^,^16^,^17^, tubules^18^,^19^, nuclei^19^,^20^, and lymphocytes^21^, (ii) cancer grading^19^,^22^, (iii) correlation of quantitative histologic image features and molecular features of breast cancer aggressiveness^23^, and (iv) identification of histologic image features that are predictive of breast cancer outcome and survival^24^.

These previous approaches have typically limited their analysis to only small portions of tissue or tissue microarrays (TMAs) as opposed to larger whole slide images. Basavanhally *et al*.^22^, looked at the problem of computerized Bloom-Richardson grading of estrogen receptor positive breast cancers within manually defined regions of interest on whole slide images. While some approaches have looked at the problem of classifying images as either containing cancer or not^25^,^26^, no approach that we are aware of has tackled the problem of automated delineation of invasive carcinoma on whole slide images.

Neural network learning refers to a class of machine learning methods that is gaining popularity in histopathology image analysis^13^,^17^,^27^,^28^,^29^,^30^,^31^,^32^,^33^. A neural network is composed of artificial neurons that are arranged in layers and interchange information through connections. In recent years, neural network models comprising thousands of neurons arranged in several layers have been shown to perform exceptionally well in computer vision and pattern analysis tasks^34^,^35^,^36^,^37^. Multi-level neural network learning approaches have recently acquired the name “deep learning” because of their multi-layer architecture. These networks are able to learn multiple levels of image representation to model complex non-linear relationships in the data, discovering more abstract and useful features that make it easier to extract useful information for high-level decision tasks such as segmentation, classification and prediction^38^,^39^,^40^. Because of the large number of parameters involved, deep learning methods require a large number of labeled training exemplars in order to be optimally trained. In problems where large numbers of training exemplars are available, deep learning methods have shown impressive prediction results, often outperforming state-of-the-art classification methods^36^,^37^,^38^. The advent of digitized whole pathology slides and the concomitant increase in the number of publicly available large histopathology image databases, such as The Cancer Genome Atlas, has made digital pathology a good candidate for the application of deep learning based classification models^13^,^17^,^27^,^28^,^29^,^30^,^31^,^32^,^33^.

In this study, we present a classification approach for detecting presence and extent of invasive breast cancer on whole slide digitized pathology images using a ConvNet classifier^38^,^41^,^42^. To ensure robustness of the classifier to variations in slide preparation, staining, and choice of scanning platform, we trained and validated the classifier with a large number of training exemplars drawn from three different institutions. Additionally the classifier was also independently evaluated on a large number of pathologic and normal cases drawn from The Cancer Genome Atlas (TCGA) and University Hospitals Case Medical Center. The goal of this study was to quantitatively evaluate the accuracy and robustness of a deep learning based machine classifier to automatically identify the extent of invasive breast cancer on digitized whole slide images.

## Results

### Quantitative evaluation for automatic invasive breast cancer detection

**Figure 1:**
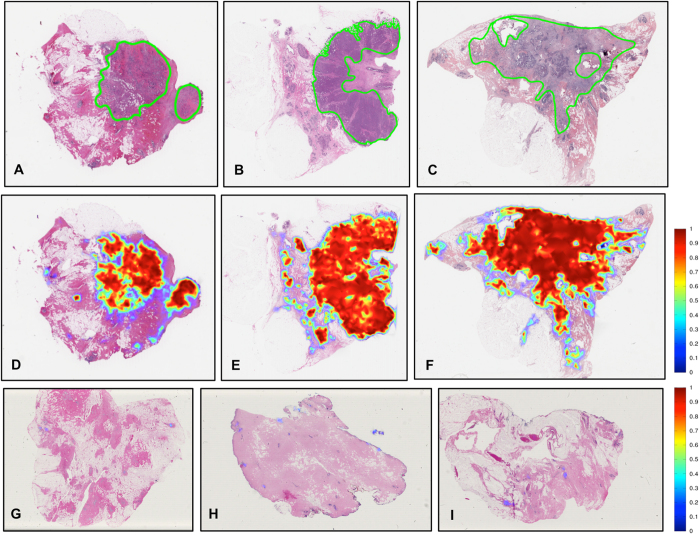
(**A**–**C**) Example whole-slide images from test TCGA data cohort with ground truth annotations from pathologists, (**D**–**F**) the corresponding region predictions produced by the ConvNet classifier and (**G**–**I**) region predictions for whole-slide images from the test NC data cohort of normal breast tissue without cancer.

Table 1 shows the detection performance of the ConvNet classifier trained with data from Hospital of the University of Pennsylvania (HUP) and University Hospitals Case Medical Center/Case Western Reserve University (UHCMC/CWRU) in terms of mean and standard deviation of Dice coefficient, positive predictive value (PPV), negative predictive value (NPV), true positive rate (TPR), true negative rate (TNR), false positive rate (FPR) and false negative rate (FNR) for the validation data set, in turn comprised of the TCGA and the NC cohorts. Figure 1 shows some representative slide images from the validation data set. Figure 1A–C depict the ground truth annotations from the pathologists on three whole-slide images from the TCGA data cohort and Fig. 1D–F represent the automatic predictions of the fully-trained ConvNet classifier as a probability map of invasive breast cancer, with the color bar reflecting the probability values, high probability values reflected in red colors and low probability values in blue colors. Finally, three example slides without any malignant pathology and part of the NC cases are illustrated in Fig. 1G–I. As may be seen in Fig. 1G–I, the ConvNet classifier did not identify any regions as having invasive breast cancer.

### Robustness and reproducibility analysis inside heterogeneous histopathology slides

A detailed analysis by subgroups of only a type of invasive breast cancer (i.e. IDC or ILC) and mixture of invasive and other types of *in situ* lesions (e.g. DCIS and LCIS) is presented in Table 2 for each of *ConvNet*_*HUP*_ and *ConvNet*_*UHCMC/CWRU*_ classifiers. Each of *ConvNet*_*HUP*_ and *ConvNet*_*UHCMC/CWRU*_ was trained with one of either the HUP or the UHCMC/CWRU cohorts. The quantitative performance results for both classifiers, *ConvNet*_*HUP*_ and *ConvNet*_*UHCMC/CWRU*_, on the validation CINJ data cohort (*ConvNet*_*HUP*_: Dice = 0.6771, PPV = 0.6464, NPV = 0.9709; *ConvNet*_*UHCMC/CWRU*_: Dice = 0.6596, PPV = 0.6370, NPV = 0.9663) are similar. The results in Table 2 are also arranged according to the type of tumors in the sample (mixture or only invasive) and reveal that our method has better performance when the whole-slide images have only one type of invasive tumor (*ConvNet*_*HUP*_: Dice = 0.7578, PPV = 0.7462, NPV = 0.9654; *ConvNet*_*UHCMC/CWRU*_: Dice = 0.7596, PPV = 0.7462, NPV = 0.9614).

**Figure 2:**
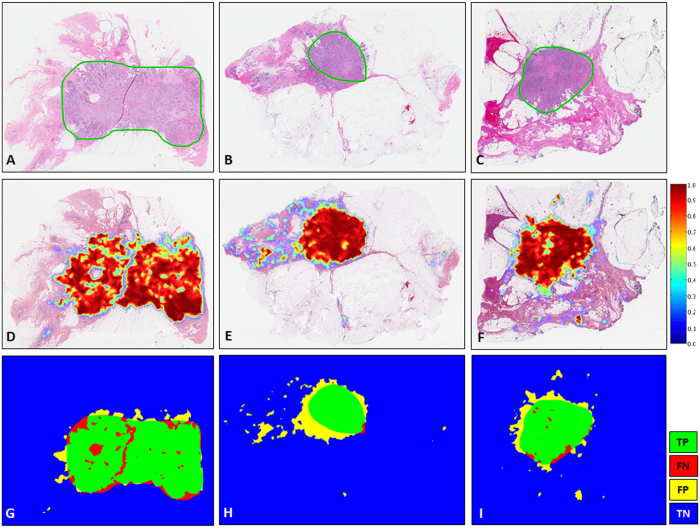
Example results for the *ConvNet*_*HUP*_ classifier on the CINJ validation data cohort. The probability map predicted by the *ConvNet*_*HUP*_ classifier (second row, (**D**–**F**)) was compared against ground truth annotations by a pathologist (first row (**A**–**C**)). The third row shows the evaluation results of the *ConvNet*_*HUP*_ classifier in terms of TP (green), FN (red), FP (yellow), and TN (blue) regions.

**Figure 3:**
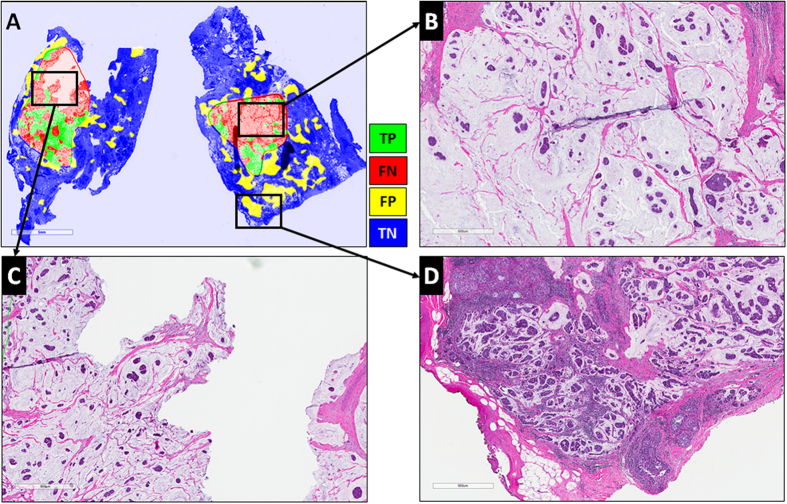
Whole-slide image from CINJ validation data cohort diagnosed with a rare type of IDC: mucinous carcinoma of the breast. (**A**) The comparison between the ground truth annotations and the predictions from the *ConvNet*_*HUP*_ classifier reveal both FN (red) and FP (yellow) errors. (**B**,**C**) Most of the FN regions, i.e. tissues wrongly labeled as non-invasive tumor, correspond to mucinous carcinoma, whilst (**D**) most of FP regions, i.e. tissues wrongly predicted as invasive tumor, are actually invasive mucinous carcinoma that was not included in the annotations by the pathologist.

**Figure 4:**
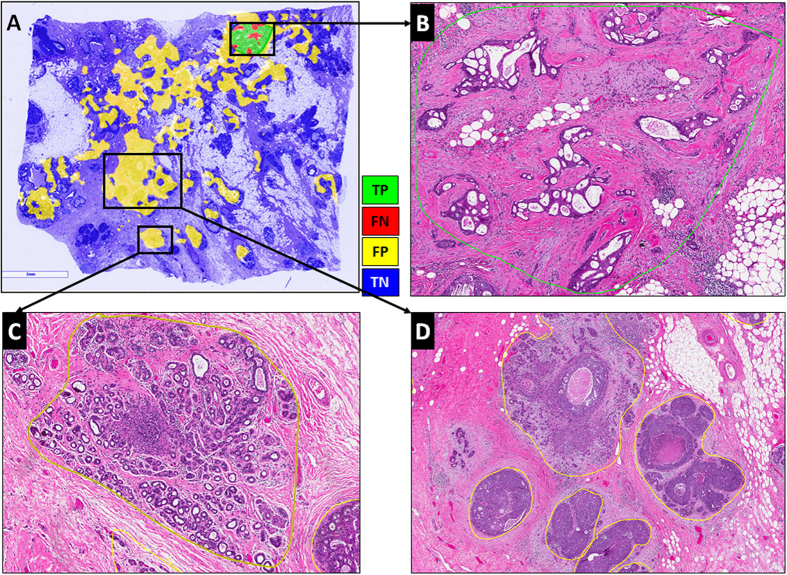
The most challenging whole-slide image in the CINJ validation cohort achieved the poorest performance via the *ConvNet*_*HUP*_ classifier with (**A**) many FP regions and a Dice coefficient of 0.0745. (**B**) Some of the FN errors are due to the confounding morphologic attributes of the tumor, arising due to a mixing of IDC with fat cells and irregular, infiltrating looking cribriform glands with DCIS. The FP regions appear to be primarily be due to (**C**) sclerosing adenosis, and (D) DCIS surrounded by IDC.

Figure 2 illustrates representative examples of whole slide images from the validation CINJ data cohort, involving only a single type of invasive tumor. The detection results obtained via *ConvNet*_*HUP*_ classifier were compared against the ground truth annotations. Some cases from the CINJ validation data cohort where the *ConvNet*_*HUP*_ classifier resulted in a poor detection performance are illustrated in Figs 3 and 4. The true-positives (TP), true-negatives (TN), false-positives (FP) and false-negatives (FN) regions, based on the predictions of the *ConvNet*_*HUP*_ classifier, are illustrated in green, blue, yellow and red respectively. Figure 3 shows a case of mucinous (colloid) carcinoma, which is a rare type of invasive ductal carcinoma with a very low prevalence (2–3% of the total invasive breast cancer cases)^43^. Figure 4 depicts a challenging case, which is composed of a mixture of invasive and *in situ* carcinoma elements.

### Correspondence and reproducibility analysis among different classifiers and data cohorts

**Figure 5:**
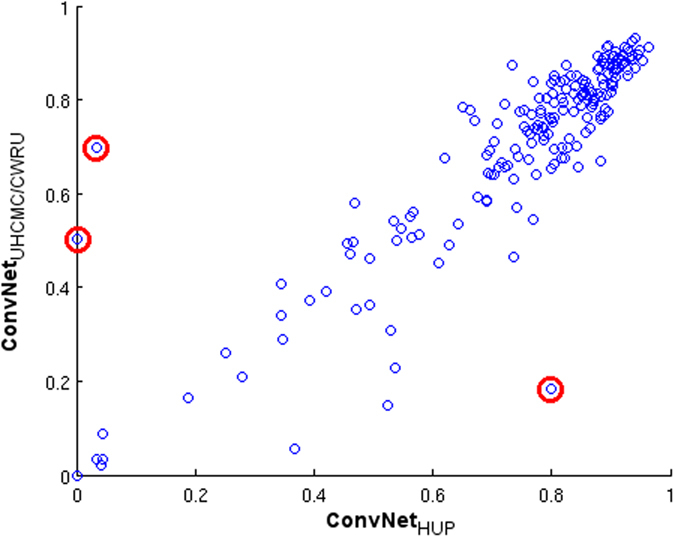
Agreement plot of the Dice coefficient for the *ConvNet*_*HUP*_ (X-axis) and *ConvNet*_*UHCMC/CWRU*_ (Y-axis) classifiers for each slide (blue circles) in the TCGA cohort. The slides with higher disagreement are identified with red circles (see Fig. 6).

**Figure 6:**
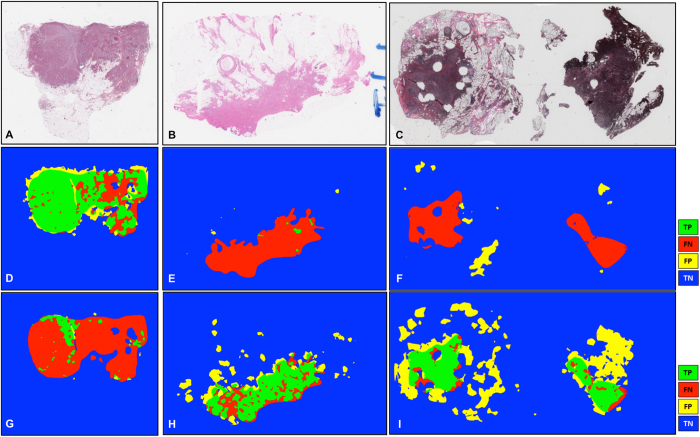
(**A**–**C**) Slides from the TCGA cohort which revealed disagreement between the predictions of the *ConvNet*_*HUP*_ and *ConvNet*_*UHCMC/CWRU*_ classifiers. The predictions of the (**D**–**F**) *ConvNet*_*HUP*_ and (**G**–**I**) *ConvNet*_*UHCMC/CWRU*_ classifiers were compared against the ground truth annotations in terms of TP (green), FN (red), FP (yellow) and TN (blue) regions.

Table 3 illustrates the performance measures for the *ConvNet*_*HUP*_ and *ConvNet*_*UHCMC/CWRU*_ classifiers on the TCGA and NC testing sets. The consistency of the predictions of both models is estimated by calculating the correlation coefficient, *r*, between the performance measures obtained for each of *ConvNet*_*HUP*_ and *ConvNet*_*UHCMC/CWRU*_. On the TCGA cohort, the correlation coefficient in Dice coefficient for *ConvNet*_*HUP*_ and *ConvNet*_*UHCMC/CWRU*_ was *r* = 0.8733, reflecting a high degree of concordance. Figure 5 shows a scatter plot where the X axis corresponds to the Dice coefficient of the predictions generated by *ConvNet*_*HUP*_ and the Y axis corresponds to the Dice coefficient of the predictions generated by *ConvNet*_*UHCMC/CWRU*_, each dot corresponds to a slide sample from the TCGA data cohort. The scatter plot in Fig. 5 reveals a well-defined cluster with most cases aggregating in the upper-right corner. The scatter plot suggests that both the *ConvNet*_*HUP*_ and *ConvNet*_*UHCMC/CWRU*_ classifiers have a high degree of agreement in their predictions of the presence and extent of invasive tumor regions. Figure 5 also helps identify cases (red circles) where both the *ConvNet*_*HUP*_ and *ConvNet*_*UHCMC/CWRU*_ disagreed in their predictions. Figure 6 showcases the test images where the classifiers tended to disagree. A closer inspection of these cases suggested, suggests that the lack of concordance is primarily in those cases where the staining characteristics substantially deviate from the staining in the cases in the training cohorts. Figure 6A,B illustrate a couple of slides characterized by low levels of hematoxylin and high levels of eosin. The slide shown in Fig. 6C illustrates an example of a “black discoloration artifact” due to air bubbles on the slide, a common problem when the slide has been in storage for a long time. Usually, these cases are not appropriate for diagnosis and a pathologist would probably reject them in a quality control process ordering for another slide to be cut from the tissue sample.

Despite these special cases of disagreement caused by staining issues, both the *ConvNet*_*HUP*_ and *ConvNet*_*UHCMC/CWRU*_ classifiers yielded similar predictions and performance. However, the *ConvNet*_*HUP*_ classifier appears to have a slightly higher confidence interval associated with the Dice and PPV performance measures. On the other hand, NPV and TNR from both classifiers show high mean values with very small standard deviation. Similarly on the NC data cohort, which is exclusively composed of normal breast samples, both the *ConvNet*_*HUP*_ and *ConvNet*_*UHCMC/CWRU*_ classifiers exhibited a very high mean TNR and a very low FPR, with very low associated standard deviation. This appears to suggest that both classifiers are able to confidently and consistently reject non-invasive tissue regions.

**Figure 7:**
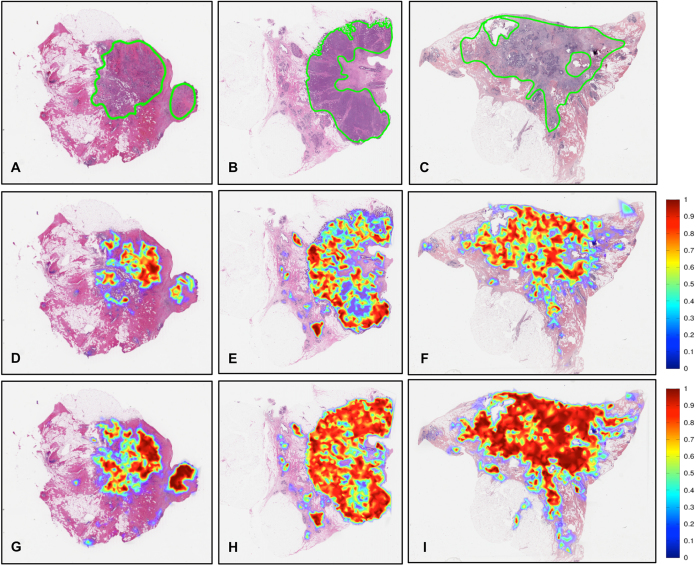
(**A**–**C**) Example whole-slide images from the TCGA data cohort with corresponding ground truth annotations. The probability maps generated by the *ConvNet*_*UHCMC/CWRU*_ and *ConvNet*_*HUP*_ classifiers are shown in panels (**D**–**F**,**G**–**I**) respectively.

**Figure 8:**
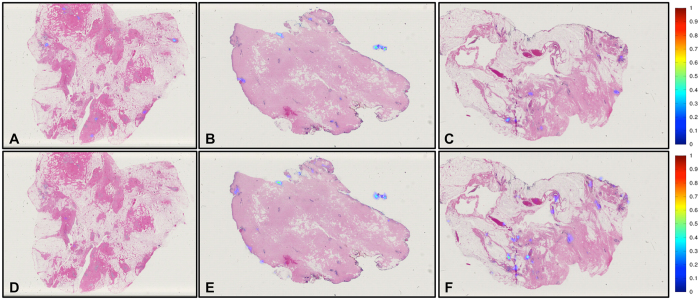
The probability maps obtained via the *ConvNet*_*UHCMC/CWRU*_ and *ConvNet*_*HUP*_ classifiers on whole-slide images of normal breast sections from the UHCMC/CWRU and NC data cohorts are shown in panels (**A**–**C**,**D**–**F**) respectively.

Example results of the predictions from the *ConvNet*_*HUP*_ and *ConvNet*_*UHCMC/CWRU*_ classifiers on the TCGA and NC test data sets are presented in Figs 7 and 8. While both the *ConvNet*_*HUP*_ and *ConvNet*_*UHCMC/CWRU*_ classifiers tend to produce consistent predictions, the ConvNet classifier, which was trained using the complete training data set, had the best overall performance (Fig. 1).

## Discussion

The experimental results show that the method is able to detect invasive breast cancer regions on whole slide histopathology images with a high degree of precision, even when tested on cases from a cohort different to the one used for training. The most challenging cases for the method were slides where invasive breast cancer was mixed in with *in situ* disease (which is not surprising and could be reduced by training a more complex network that included examples of these precursor lesions).

An important part of the experimental setup was the analysis of the detection sensitivity of the method to the data used for training. The results show that the classifiers trained with two different data cohorts, HUP and UHCMC/CWRU, exhibit highly correlated performance measures (*r* ≥ 0.8) over the independent TCGA test data cohort (see Table 3). Despite this, there are some differences in the prediction performance of the two classifiers, possibly suggesting “batch effects”^44^, that originated from the process of ground truth annotation or slide digitization. This is illustrated in Figs 5 and 6, which show representative slides with artifacts due to problems in the histotechnique process. The method shows a very low false positive rate, as evidenced by the results in the NC cohort (*ConvNet*_*HUP*_: FPR = 0.0284; *ConvNet*_*UHCMC/CWRU*_: FPR = 0.0454), which comprised only normal breast sections. The performance of the ConvNet improved as the number of training samples increased, i.e. the ConvNet classifier trained with both the HUP and UHCMC/CWRU data cohorts yielded the best overall performance (Table 1 and Fig. 1).

The ConvNet was used as a patch-based classifier. We addressed the tissue classification task through a learned feature approach instead of a hand-crafted feature approach^13^,^17^,^27^,^29^,^38^,^42^. However, any statistical or machine learning classifier could be used in combination with a set of hand-crafted features for tissue classification. For instance, in addition to successful deep learning methods (i.e. ConvNets and Autoencoders) applied in histopathology image analysis^13^,^17^,^27^,^28^,^29^, a set of hand-crafted features (color/intensity features, texture features, graph-based features, etc.) and machine learning methods (random forests and support vector machines) could and have been applied to histopathology image analysis^33^,^45^,^46^,^47^,^48^. We did a comparative analysis with some of these visual features used in histopathology image analysis against three different ConvNet architectures. The ConvNet classifiers showed better performance in our patch-based image classification task. These results are presented in subsection: Invasive Breast Cancer Tissue Detection in Whole-Slide Images.

Our study did, however, have its limitations. There are some subtypes of invasive breast cancers that our method is not able to detect in a precise way such as the rare special histologic subtype mucinous carcinoma that comprises around 3% of the invasive breast cancers. In fact, in the test data set there are two cases similar to Fig. 3, with mucinous carcinoma that were not detected. Another limitation is that some *in situ* breast cancer regions were incorrectly classified as invasive breast cancer, *in situ* disease is different from invasive cancer. However, the reporting of the presence of both invasive and *in situ* carcinoma is a critical part of a diagnostic pathology workup. It is worth noting though that our approach was able to achieve a very high level of accuracy in terms of rejecting non-invasive tissue regions (normal controls) as not being cancer. Exemplars of DCIS and LCIS could, in future work, be included as part of an expanded learning set, as it would not doubt improve the classification performance and generalizability of the model. Additionally and as part of future work, the learning set could be expanded to include other rare variants of invasive ductal carcinoma, such as mucinous invasive carcinomas.

Batch effects are one of the main sources of variation in evaluating the performance of automated machine learning approaches. These batch effects include stain variability due to different histology protocols from different pathology labs and variations in the digitization process on account of the use of different slide scanners^44^. Our results suggest a slight batch effect with two different data cohorts (*ConvNet*_*HUP*_ and *ConvNet*_*UHCMC/CWRU*_). Results of Table 2 appears to suggest that the differences between both classifiers is related more to the number of samples employed for training each of the classifiers (HUP, *N* = 239, and UHCMC/CWRU, *N* = 110) and possibly less related to the constitution of the different histologic subtypes within the training cohorts. However, the use of all available training data (HUP and UHCMC/CWRU) results in a more confident, accurate and robust ConvNet classifier. Clearly, increasing the training data set size and diversity results in a better and more robust algorithm. ConvNet also performs better when a case has only a single morphologic pattern of invasive breast cancer in the whole slide images. Cases with a mixture of invasive and *in situ* breast cancer resulted in a reduction in the overall accuracy of the ConvNet classifier (*in situ* tumors may be incorrectly classified as invasive carcinoma). One way of potentially reducing batch effects is to apply color normalization on the digitized images prior to training or application of the ConvNet classifier. To reduce false positive classification errors we are exploring the expansion of the current two class ConvNet classifier into a multiclass predictor. This will allow for the ConvNet classifier to explicitly deal with the detection of additional subtypes of invasive and *in situ* breast cancers.

One interesting aspect of our work is that the trained ConvNet classifier can be easily integrated into other computational frameworks such as automated tumor grading of ER+ breast cancer subtypes in histopathology images^22^. Our automated invasive cancer detection algorithm could thus pave the way for creation of decision support tools for breast cancer diagnosis, prognosis and theragnosis for use by the pathology community. Future studies will address these opportunities. Additionally follow on work will need to systematically compare the approach presented in this paper with state of the art visual features and machine learning approaches that have been previously applied to the problem of histopathology image analysis.

In conclusion, we presented an automatic invasive breast cancer detection method for whole slide histopathology images. Our study is unique in that it involved several hundred studies from multiple different sites for training the model. Independent testing of the model on multi-site data revealed that the model was both accurate and robust. This method can be applied to large, digitized whole slide images to detect invasive tissue regions, which could be integrated with other computerized solutions in digital pathology such as tumor grading.

## Methods

### Ethics Statement

Data analysis was waived review and consent by the IRB board, as all data was being analyzed retrospectively, after de- identification. All experimental protocols were approved under the IRB protocol No. 02-13-42C with the University Hospitals of Cleveland Institutional Review Board, and all experiments were carried out in accordance with approved guidelines.

### Patients and Data Collection

This study involved images from five different cohorts from different institutions/pathology labs in the United States of America and TCGA^49^,^50^. The five cohorts were used for training, validation and independent testing of our method. The training data set had 349 estrogen receptor-positive (ER+) invasive breast cancer patients, of which 239 were from Hospital of the University of Pennsylvania (HUP), and 110 from University Hospitals Case Medical Center/Case Western Reserve University (UHCMC/CWRU). Patients from the HUP cohort ranged in age between 20 and 79 (average age 55 ± 10). In the UHCMC/CWRU cohort, the patient ages ranged from 25 to 81 (average age 58 ± 10). The validation data set contained 40 ER+ invasive breast cancer patients from the Cancer Institute of New Jersey (CINJ). The test data set was composed of two distinct subsets of positive and negative controls. For the test data set, we accrued a set of 195 ER+ invasive breast cancer cases from TCGA, ages ranging from 26 to 90 (average age 57 ± 13). For the negative controls (NC) in the test data set, we used normal breast tissue sections taken from uninvolved adjacent tissue from 21 patients diagnosed with invasive ductal carcinoma from UHCMC/CWRU, Cleveland, OH. Patient specific information pertaining to race, tumor grade, and outcome were not explicitly recorded for this study.

Hematoxylin and eosin (H&E) slides from all the various training, validation and testing cohorts (HUP, CINJ, UHCMC/CWRU, TCGA) were independently reviewed by four expert pathologists (NS, JT, MF, HG) to confirm the presence of at least one type of invasive breast cancer tumor. The normal control H&E slides were reviewed by one pathologist (HG). Tumors were categorized into one of the following histological types: invasive carcinoma were categorized as either invasive ductal carcinoma (IDC) or invasive lobular carcinoma (ILC), while pre-invasive carcinoma was categorized as ductal carcinoma *in situ* (DCIS) or lobular carcinoma *in situ* (LCIS). Only those cases were considered in our study where at least two pathologists concurred on the diagnosis.

### Slide Digitization and Pathologists Ground Truth

H&E stained histopathology slides were digitized via a whole-slide scanner at 40x magnification for this study. An Aperio Scanscope CS scanner was used to digitize cases from the HUP, CINJ and TCGA cohorts. The Ventana iCoreo scanner was used for scanning the UHCMC/CWRU and NC data cohorts. 40x magnification corresponds to Aperio’s slides at 0.25 *μ*m/pixel resolution and to Ventana’s slides at 0.23 *μ*m/pixel.

Expert pathologists provided the ground truth annotations of invasive breast cancer regions for all the data cohorts (HUP, CINJ, UHCMC/CWRU, TCGA). The region annotations were obtained via manual delineation of invasive breast cancer regions by expert pathologists using the ImageScope v11.2 program from Aperio and the Ventana Image Viewer v3.1.4 from Ventana. To alleviate the time and effort required to create the ground truth annotations for extent of invasive breast cancer, the pathologists were asked to perform their annotations at 2x magnification or less. All whole-slide images previously sampled at 40x were thus subsequently downsampled (by a factor of 16:1) to a resolution of 4 *μ*m/pixel.

**Figure 9:**
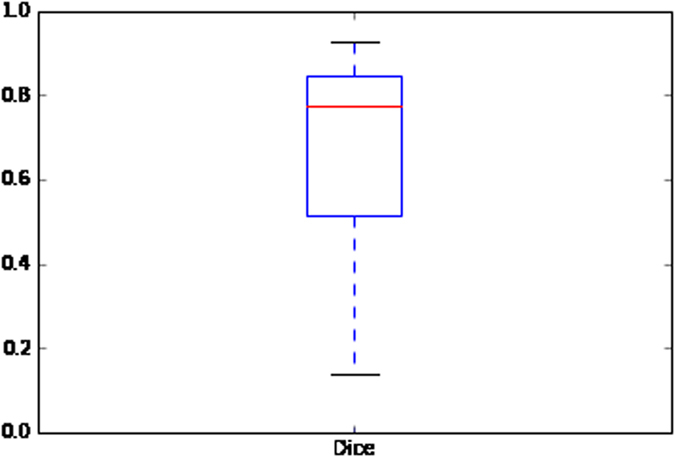
Dice coefficient between pathologist annotations for the CINJ data cohort (*N* = 40).

In order to analyze the agreement between expert pathologists, the Dice coefficient and Cohen’s Kappa coefficient were calculated between NS + MF and HG manual delineations. The Cohen’s Kappa coefficient was determined to be *κ* = 0.748^51^, in turn reflecting good agreement between the experts^52^. In addition, the Dice coefficient was calculated to measure the overlap between the cancer annotations between NS + MF and HG delineations and was determined to be *DSC* = 0.6685^53^. Figure 9 below depicts the Dice coefficient dispersion between expert pathologists. Figure 9 shows that the DSC measure is not a Gaussian distribution and has a median value equal to 0.7764. The DSC agreement was found to be greater than 0.7 for a majority of the images studied, where good agreement is typically defined as when agreement is greater than 60%.

### Invasive Breast Cancer Tissue Detection in Whole-Slide Images

**Figure 10:**
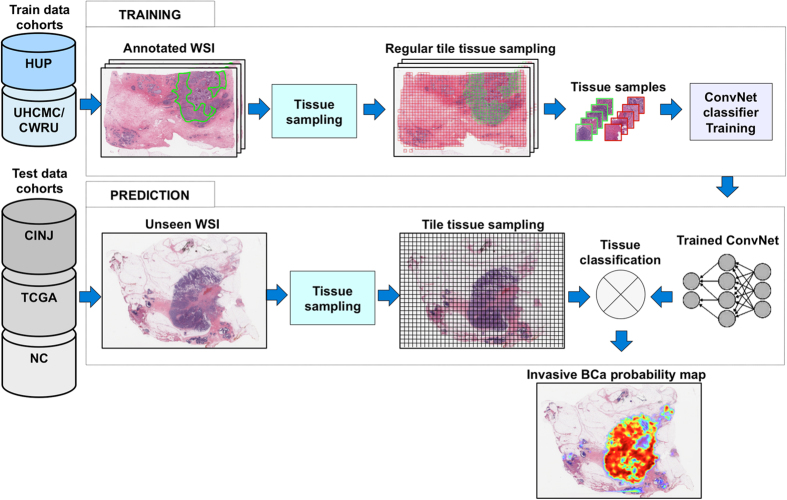
Overview of the process of training and testing of the deep learning classifiers for invasive breast cancer detection on whole-slide images. The training data set had 349 ER+ invasive breast cancer patients (HUP N = 239, UHCMC/CWRU N = 110). The validation data set contained 40 ER+ invasive breast cancer patients from the Cancer Institute of New Jersey (CINJ). The test data set was composed of 195 ER+ invasive breast cancer cases from TCGA and 21 negative controls (NC).

**Figure 11:**
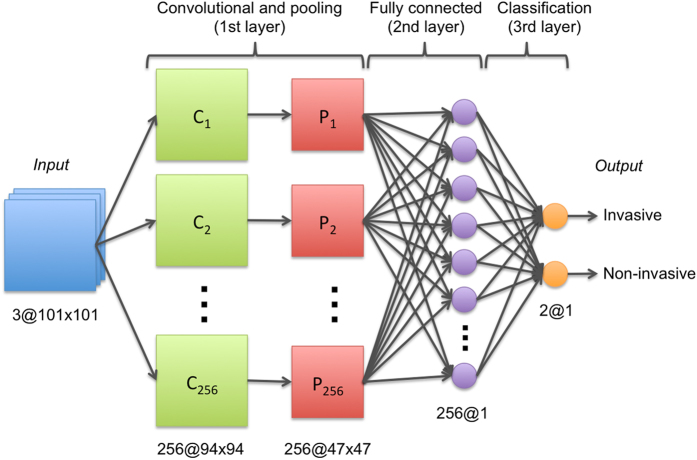
3-layer ConvNet architecture.

Our deep-learning based approach for detection of invasive breast cancer on whole-slide images is illustrated in Fig. 10. The approach comprises three main steps: (i) tile tissue sampling, (ii) tile pre-processing, and (iii) convolutional neural network (ConvNet) based classification. In this work, a tile is a square tissue region with a size of 200 × 200 *μ*m. The tile tissue sampling process involves extraction of square regions of the same size (200 × 200 *μ*m), on a rectangular grid for each whole-slide image. Only tissue regions are invoked during the sampling process and any regions corresponding to non-tissue within the background of the slide are ignored. The first part of the tile pre-processing procedure involves a color transformation from the original Red-Green-Blue color space representation to a YUV color space representation. A color normalization step is then applied to the digitized slide image to get zero mean and unit variance of the image intensities, and to remove correlations among the pixel intensity values. Tiles extracted from new whole-slide images, different from the ones used for training, are preprocessed using the same mean and standard deviation values in the YUV color space learned during training. The ConvNet classifier^41^,^42^, was trained using a set of image tiles extracted from invasive (positive examples) and non-invasive (negative examples) tissue regions, annotated on whole slide digitized images by expert pathologists. Positive examples were identified as those in which the detected cancer regions had a minimum of 80% overlap with the manual annotations of the expert pathologists. Three different ConvNet architectures were evaluated using the training data: 1) a simple 3-layer ConvNet architecture, 2) a typical 4-layer ConvNet architecture, and 3) a deeper 6-layer ConvNet architecture. The 3-layer ConvNet architecture is constituted as follows, the first layer is the convolutional and pooling layer and the second is a fully connected layer, where each layer has 256 units (or neurons). The third is the classification layer with two units as outputs, one for each class (invasive and non-invasive), corresponding to a value between zero and one. The 4-layer ConvNet architecture is comprised of an initial convolutional and pooling layer with 16 units, followed by a second convolutional and pooling layer with 32 units, the third layer is a fully connected layer with 128 units, and the final classification layer comprises two units as class outputs (invasive and non-invasive). The 6-layer ConvNet architecture comprises four convolutional and pooling layers with 16 units, a fully connected layer with 128 units, and a final classification layer with two units as class outputs (invasive and non-invasive). The 3-layer ConvNet resulted in the best performance and hence was selected as the model of choice for all subsequent experiments (Fig. 11). The implementation of the ConvNets classifier was performed using Torch 7, a scientific computing framework for machine learning^54^.

The ConvNet classifier was trained with images from HUP and UHCMC/CWRU. The training set comprised a large number of cases manually annotated by pathologists, i.e. 349 cases (239 from HUP and 110 from UHCMC/CWRU). The validation data cohort was the smaller data set with manual annotations from pathologists of invasive tumors (CINJ, N = 40), and the testing data sets were: a publicly available data set with invasive tumors (TCGA, N = 195) and normal control cases without breast cancer (NC, N = 21). Our training set comprised a total of 344,662 patches, of which 91,952 were from the positive class (invasive) and 252,710 were from the negative class (non-invasive). We applied data augmentation only to the positive class, the positive class being the minority class in terms of number of samples. The data augmentation process for the positive class comprised of duplicating the number of patches with artificial rotations and mirroring of patches. The weights were randomly initialized and updated during the training stage by using the stochastic gradient descent algorithm. This strategy was used to “learn” the weights (features) of the network from the training set. The number of epochs to train the ConvNets classifiers was 25. The mini-batch size was 32. The remaining parameters for the ConvNet classifier were tuned during the training process. These parameters included the learning rate, learning rate decay, non-linear function and pooling function. The optimal parameter configuration was determined to be 1*e*^−3^, 1*e*^−7^, ReLU and L2-norm, respectively. The best parameter configuration of the classifier was identified using the average area under the ROC curve (AUC) calculated over all slides in the CINJ data cohort, N = 40. The CINJ data cohort was used as the validation data set because it is the smaller pathological data set with manual independent annotations from 3 different pathologists of invasive tumors. The AUC is a non-biased classification measure that allows for the evaluation of classification performance independent of a fixed threshold. In this work classification performance was evaluated over all the image tiles extracted from all the whole-slide images in the CINJ data cohort, tiles that correspond to either invasive or non-invasive tissue classes. Table 4 presents a comparison between the ConvNet classifiers and state of the art handcrafted visual features (color, shape, texture and topography) used in histopathology image analysis. The classification results associated with these handcrafted features is lower compared to the ConvNet classifier and also results in more variability. The comparative evaluation helped identify the ConvNet classifier with the best classification performance and simplest configuration (Avg. AUC = 0.9018 ± 0.0093) for the subsequent experiments involving the independent test set.

### Method evaluation

We evaluated the accuracy of the ConvNet classifier in whole slide images by comparing the predictions of invasive regions in the test data set against the corresponding ground-truth regions annotated by expert pathologists. The test data sets included the slides in the TCGA and NC cohorts. A quantitative evaluation was performed by measuring the Dice coefficient, positive predictive value (PPV), negative predictive value (NPV), true positive rate (TPR), true negative rate (TNR), false positive rate (FPR) and false negative rate (FNR) across all the test slides. These measures were evaluated for each whole-slide image and the mean and standard deviation in performance measures were calculated for each test data cohort.

In addition to training the ConvNet classifier with the full training data set (HUP and UHCMC/CWRU), two additional classifiers were trained using, in each case, one of the training cohorts: *ConvNet*_*HUP*_ trained with the HUP cohort and *ConvNet*_*UHCMC/CWRU*_ trained with the UHCMC/CWRU cohort. The motivation was to analyze the sensitivity of the classifier to the training data sets. Both *ConvNet*_*HUP*_ and *ConvNet*_*UHCMC/CWRU*_ were evaluated on both the validation (CINJ cohort) and test data sets (TCGA and NC cohorts) to analyze how and where their predictions diverged. Specifically we measured the correlation coefficient r between the prediction performance measures for *ConvNet*_*HUP*_ and *ConvNet*_*UHCMC/CWRU*_ across all slides in each test cohort.

## Additional Information

**How to cite this article:** Cruz-Roa, A. *et al*. Accurate and reproducible invasive breast cancer detection in whole-slide images: A Deep Learning approach for quantifying tumor extent. *Sci. Rep.* **7**, 46450; doi: 10.1038/srep46450 (2017).

**Publisher's note:** Springer Nature remains neutral with regard to jurisdictional claims in published maps and institutional affiliations.

## Figures and Tables

**Table 1:** Performance measures for the ConvNet classifier on the TCGA (pathological, N = 195) and NC (normal, N = 21) data cohorts.

Data set	Dice	PPV	NPV	TPR	TNR	FPR	FNR
TCGA	0.7586 ± 0.2006	0.7162 ± 0.2204	0.9677 ± 0.0511	0.8691 ± 0.1582	0.9218 ± 0.0764	0.0782 ± 0.0764	0.1309 ± 0.1582
NC	N/A	N/A	1 ± 0	N/A	0.9964 ± 0.0110	0.0036 ± 0.0110	N/A

The measures included Dice, PPV, NPV, TPR, TNR, FPR and FNR. Note that for the normal cases considered, not all the performance measures are shown because the NC data cohort did not have cancer annotations.

**Table 2:** Performance of the *ConvNet*_*HUP*_ and *ConvNetU*_*HCMC/CWRU*_ classifiers on the CINJ data cohort in terms of means and standard deviation of Dice coefficient, PPV and NPV.

Group	N	Dice	PPV	NPV
*ConvNet*_*HUP*_				
All cases	40	0.6771 ± 0.2445	0.6464 ± 0.2870	0.9709 ± 0.0350
Only invasive	19	0.7578 ± 0.2166	0.7462 ± 0.2480	0.9654 ± 0.0355
Mixture	21	0.6041 ± 0.2501	0.5560 ± 0.2953	0.5560 ± 0.2953
*ConvNet*_*UHCMC/CWRU*_				
All cases	40	0.6596 ± 0.2527	0.6370 ± 0.2941	0.9663 ± 0.0421
Only invasive	19	0.7596 ± 0.2074	0.7499 ± 0.2423	0.9614 ± 0.0440
Mixture	21	0.5691 ± 0.2602	0.5348 ± 0.3045	0.9708 ± 0.0409

The results in Table 2 are organized in terms of all cases in the CINJ cohort (N = 40), a subset of the CINJ cohort with invasive breast cancer alone (N = 19), and a mixture of invasive and other *in situ* subtypes of breast cancer (N = 21).

**Table 3:** Comparison and correlation of the *ConvNet*_*UHCMC/CWRU*_ and *ConvNet*_*HUP*_ classifiers in terms of Dice, PPV, NPV, TPR, TNR, FPR and FNR.

	Dice	PPV	NPV	TPR	TNR	FPR	FNR
TCGA							
*ConvNet*_*HUP*_	0.7494 ± 0.2071	0.7071 ± 0.2254	0.9658 ± 0.0514	0.8600 ± 0.1705	0.9188 ± 0.0805	0.0812 ± 0.0805	0.1400 ± 0.1705
*ConvNet*_*UHCMC/CWRU*_	0.7068 ± 0.2061	0.6464 ± 0.2188	0.9629 ± 0.0584	0.8676 ± 0.1706	0.8880 ± 0.0824	0.1120 ± 0.0824	0.1324 ± 0.1706
*r*	0.8733	0.9258	0.8109	0.6345	0.8055	0.8055	0.6345
NC							
*ConvNet*_*HUP*_	N/A	N/A	1 ± 0	N/A	0.9716 ± 0.0693	0.0284 ± 0.0693	N/A
*ConvNet*_*UHCMC/CWRU*_	N/A	N/A	1 ± 0	N/A	0.9546 ± 0.0816	0.0454 ± 0.0816	N/A
*r*	N/A	N/A	N/A	N/A	0.6876	0.6876	N/A

Note that for the normal cases considered, not all the performance measures are shown because the NC data cohort did not have cancer annotations.

**Table 4:** Comparison of ConvNet classifiers and visual features (color, shape, texture and topography) in terms of AUC.

Method	AUC
6-layer ConvNet	0.9021 +/− 0.0097
3-layer ConvNet	0.9018 +/− 0.0093
4-layer ConvNet	0.8915 +/− 0.0093
Color and intensity features^47^,^55^,^56^	0.8711 +/− 0.0947
Color histograms^47^,^56^	0.8448 +/− 0.1047
Shape Index histogram^57^	0.8444 +/− 0.1065
Haralick features^47^,^56^,^58^.	0.8385 +/− 0.0942
Topography and Graph-based features^46^,^55^,^56^	0.7998 +/− 0.1068
